# Leukocyte-Platelet-Rich Fibrin Combined With Demineralized Bovine Bone Mineral for Maxillary Sinus Augmentation: A Systematic Review and Meta-Analysis of Randomized Controlled Trials

**DOI:** 10.7759/cureus.85306

**Published:** 2025-06-03

**Authors:** Mohammad Almutairi, Sultan Z Alshammari, Dhaifallah Almutairi, Talal A Alansari, Fares Aldhafiri, Abdulaziz N Almutairi, Abdulmohsen Alazmi, Nasser B Alkandari, Maha Alkhaldi, Madhawi Alwegyan

**Affiliations:** 1 Department of Dentistry, Sulaibyia Polyclinic, Al Jahra, KWT; 2 Department of Dentistry, Saad Al-Abdullah-Block 2 Polyclinic, Al Jahra, KWT; 3 Department of Dentistry, Abdullah Al Mubarak Al Sabah Polyclinic, Farwaniya, KWT; 4 Department of Dentistry, Saad Al-Abdullah-Block 10 Polyclinic, Al Jahra, KWT; 5 Department of Dentistry, Abdulrahman Alzaid Misref West Clinic Polyclinic, Kuwait City, KWT; 6 Department of Dentistry, Al-Waha Polyclinic, Al Jahra, KWT

**Keywords:** deproteinized bovine bone mineral, histomorphometry, leukocyte-platelet-rich fibrin, maxillary sinus augmentation, prf

## Abstract

Maxillary sinus augmentation is considered the gold standard procedure for increasing bone volume to support dental implant placement. However, the added benefit of leukocyte-platelet-rich fibrin (L-PRF) when combined with deproteinized bovine bone mineral (DBBM) remains uncertain. This systematic review and meta-analysis aimed to evaluate the histomorphometric outcomes of L-PRF combined with DBBM compared to DBBM alone. A comprehensive search of PubMed, Scopus, Web of Science, and Cochrane Central was conducted from inception through May 2025 to identify randomized controlled trials (RCTs) comparing the combination of L-PRF and DBBM versus DBBM alone in patients undergoing lateral maxillary sinus augmentation. Primary outcomes included the percentage of new bone formation and residual bone graft. Secondary outcomes were the percentage of soft tissue and the Implant Stability Quotient (ISQ) at the implant loading stage. Pooled mean differences (MDs) with 95% confidence intervals (CIs) were calculated using a random-effects model. All analyses were performed using STATA MP version 18 (StataCorp LLC, College Station, TX, US). Five RCTs involving 140 patients were included. The combination of L-PRF and DBBM significantly increased new bone formation (MD = 7.07; 95% CI: 2.20 to 11.93; p < 0.001; I² = 36.86%, p = 0.07) and reduced residual graft material (MD = -7.93; 95% CI: -11.20 to -4.66; p < 0.001; I² = 0%, p = 0.91) compared to DBBM alone. In conclusion, the adjunctive use of L-PRF with DBBM enhances histomorphometric outcomes in maxillary sinus augmentation. Further high-quality, long-term RCTs with standardized protocols are needed to confirm these findings.

## Introduction and background

The posterior maxilla often poses a clinical challenge for implant placement due to limited vertical bone height following tooth extraction. This is primarily attributed to sinus pneumatization and progressive alveolar bone resorption over time [1]. Maxillary sinus augmentation has become the gold standard procedure to increase bone volume and facilitate dental implant placement in such cases. Deproteinized bovine bone mineral (DBBM) is widely used as a grafting material in sinus augmentation due to its favorable osteoconductive properties, biocompatibility, low resorption rate, and widespread availability [2]. However, despite its extensive use, DBBM lacks inherent osteoinductive and osteogenic properties, which may result in prolonged healing periods and delayed bone regeneration [3].

More recently, platelet-rich plasma (PRP), derived from autologous blood, is known to contain a high concentration of bioactive molecules such as platelets and growth factors, which are essential for regeneration and inflammation modulation [4], and has gained much interest in surgical procedures [5]. However, the adjunctive use of leukocyte-platelet-rich fibrin (L-PRF), a second-generation autologous platelet concentrate, rather than PRP, has gained much interest in sinus augmentation procedures. Sinus augmentation is a surgical procedure aimed at increasing the bone volume by adding bone graft materials. Moreover, L-PRF consists of a dense fibrin matrix enriched with platelets, leukocytes, and growth factors such as VEGF, TGF-β1, and PDGF [6], of which these components promote angiogenesis, modulate inflammation, and enhance both soft and hard tissue healing. The hypothesis is that combining L-PRF with DBBM may improve graft bioactivity, accelerate bone regeneration, and reduce the healing time [6].

Several clinical and histomorphometric studies have investigated the efficacy of this combination, but their findings remain inconsistent [7,8]. Therefore, the present systematic review and meta-analysis aims to synthesize the current evidence and provide a conclusive evaluation of the effectiveness of adding L-PRF to DBBM in maxillary sinus augmentation compared to the use of DBBM alone.

## Review

Methods and materials

We followed the Preferred Reporting Items for Systematic Reviews and Meta-Analyses (PRISMA) guidelines [9] while conducting this systematic review and meta-analysis. We also adhered to the Cochrane Handbook for Systematic Reviews of Interventions [10].

Literature Search

A comprehensive search was conducted in PubMed, Web of Science (WOS), Scopus, and the Cochrane Central Register of Controlled Trials from inception until May 2025. The search strategy included the following terms: ("maxillary sinus augmentation" OR "sinus lift" OR "posterior maxilla") AND ("platelet-rich fibrin" OR "PRF" OR "leukocyte- and platelet-rich fibrin" OR "L-PRF") AND ("deproteinized bovine bone" OR "DBBM" OR "Bio-Oss"). The detailed search strategies for each database are presented in Table 1. We included only studies published in English and limited our search to articles published from the year 2000 onward. To ensure the comprehensiveness of the review, we also manually screened the reference lists of all included studies for additional relevant publications.

**Table 1:** Detailed search strategy according to each database.

Database	Search terms	Search field	Search results
PubMed	("maxillary sinus augmentation"OR "sinus lift" OR "posterior maxilla") AND ("platelet-rich fibrin" OR "PRF" OR "leukocyte- and platelet-rich fibrin" OR "L-PRF") AND ("deproteinized bovine bone" OR "DBBM" OR "xenograft" OR "Bio-Oss")	All Fields, English	24
Cochrane	("maxillary sinus augmentation"OR "sinus lift" OR "posterior maxilla") AND ("platelet-rich fibrin" OR "PRF" OR "leukocyte- and platelet-rich fibrin" OR "L-PRF") AND ("deproteinized bovine bone" OR "DBBM" OR "xenograft" OR "Bio-Oss")	Title, Abstract	20
Web of Science	("maxillary sinus augmentation"OR "sinus lift" OR "posterior maxilla") AND ("platelet-rich fibrin" OR "PRF" OR "leukocyte- and platelet-rich fibrin" OR "L-PRF") AND ("deproteinized bovine bone" OR "DBBM" OR "xenograft" OR "Bio-Oss")	All Fields	41
Scopus	("maxillary sinus augmentation"OR "sinus lift" OR "posterior maxilla") AND ("platelet-rich fibrin" OR "PRF" OR "leukocyte- and platelet-rich fibrin" OR "L-PRF") AND ("deproteinized bovine bone" OR "DBBM" OR "xenograft" OR "Bio-Oss")	Title, Abstract, Keywords, English	36

Eligibility Criteria

The selection of studies was carried out in two stages. Initially, all retrieved records were screened based on their titles and abstracts. Those deemed potentially relevant were then subjected to a full-text review to assess eligibility according to predefined criteria. Studies were included if they involved patients undergoing maxillary sinus augmentation, with the intervention group receiving a combination of L-PRF and DBBM and the comparison group receiving DBBM alone. Only studies that reported relevant outcomes using an intention-to-treat analysis were considered. We excluded studies that did not examine the addition of L-PRF to DBBM, as well as those with unpublished data, conference abstracts, or observational designs.

Outcomes

The primary outcome of interest was the percentage change of new bone formation, with a higher percentage of bone formation indicating a favorable outcome [11]. Additionally, other studied outcomes were the percentage of residual bone, with a lower percentage indicating a favorable outcome [11], the percentage of soft tissue calculated as the amount of fibrous tissue in the maxillary sinus cavity, and the mean values of Implant Stability Quotient (ISQ) at the implant loading stage, measured for each implant from 1 to 100 [12].

Quality Assessment

Two authors independently assessed the risk of bias in all included studies using the Cochrane Risk of Bias 2 (ROB-2) tool for evaluating randomized controlled trials (RCTs) [13]. The tool uses five different domains accounting for different methodologies of bias, which include selection bias (randomization process), performance bias (deviation from intended interventions), detection bias (outcome measurements), attrition bias (missing outcome data), and reporting bias (selection of the reported results). The decisions were labeled as “high risk of bias,” “some concerns,” and “low risk of bias.” Any disagreements between the two authors were resolved via discussion with another author.

Data Extraction and Meta-Analysis

We used a standardized Excel sheet (Microsoft Corp., Redmond, WA, US) to extract the data from the included studies. The data extracted were four domains: (1) summary of the included studies, including study design, country, follow-up duration, studied outcomes, and main findings; (2) characteristics of the included patients, including the sample size of each arm, mean age, percentage of males, residual bone height, and methodology of L-PRF procedure; (3) risk of bias domains; and (4) measurements of the studied outcomes. Continuous data were extracted as the mean and standard deviation (SD) reported from each study at the latest follow-up duration. Additionally, continuous outcomes were pooled as the mean difference (MD) with its 95% confidence interval (CI) using the Der-Simonian-Laird random-effects model. Heterogeneity was assessed using the Cochrane Q test, and the I^2^ measure was determined across all studies. A p-value less than 0.05 and I^2^ value ≥ 50% were deemed as significant diversity among the included studies. The package “meta esize” and “meta forest plot” were used on STATA 18MP software (StataCorp LLC, College Station, TX, US) to pool the effect estimate and the corresponding 95% CI.

Results

Search Results

**Figure 1:**
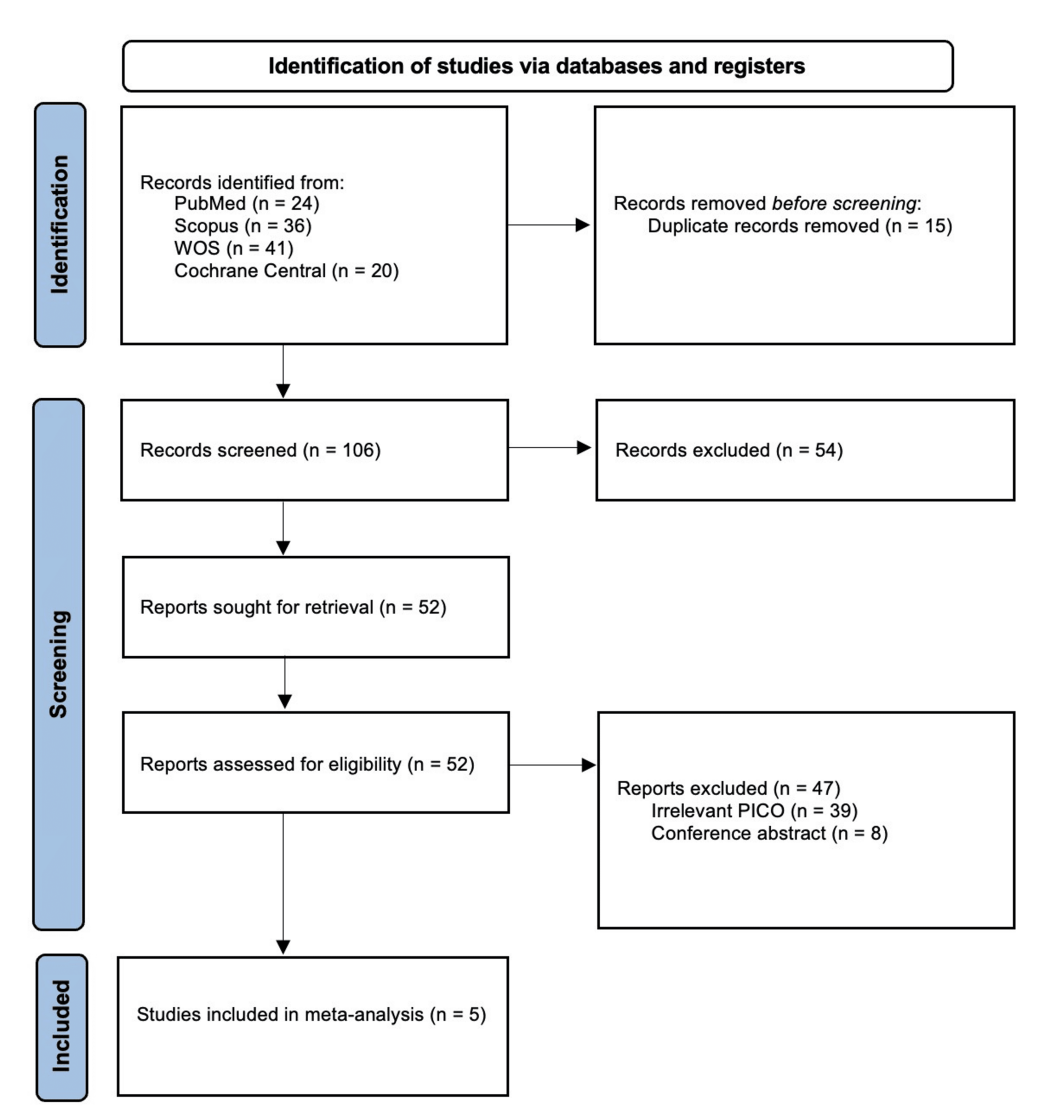
PRISMA flow chart. PRISMA: Preferred Reporting Items for Systematic Reviews and Meta-Analyses; PICO: population, intervention, comparison, outcome; WOS: Web of Science

We identified 121 citations from databases, of which 15 articles were excluded following the removal of duplicates, and finally, five RCTs [6-8,14,15] were included in the final analysis following title, abstract, and full-text screening. The study selection process is summarized in the PRISMA chart, Figure 1.

Characteristics of the Included RCTs

Five RCTs involving a total of 140 patients were included in this review. Among them, 76 patients (54.3%) received a combination of L-PRF and DBBM, while 64 patients (45.7%) received DBBM alone. The mean age across all patients was 50.42 ± 7.3 years, and 74 patients (62%) were female. In three of the included RCTs [8,14,15], the residual bone height was reported to be less than 5 mm, whereas in the remaining two studies [6,7], it was less than 4 mm. A detailed overview of the baseline characteristics and study summaries is presented in Tables 2, 3.

**Table 2:** Baseline characteristics of the included studies. L-PRF: leukocyte-platelet-rich fibrin; DBBM: deproteinized bovine bone mineral; NA: not available; SD: standard deviation

Study ID	Arms	Sample size	Age		Sex (male, n, %)	Residual bone height
			Mean	SD		
de Almeida Malzoni et al., 2023 [7]	L-PRF + DBBM4	12	54.08	10.07	10 (27.28%)	<4 mm
	L-PRF + DBBM8	12				
	DBBM alone	12				
Nizam et al., 2018 [8]	L-PRF + DBBM4	11	49.92	10.37	9 (69%)	<5 mm
	DBBM alone	11				
Pichotano et al., 2019 [6]	L-PRF + DBBM4	6	54.17	6.95	6 (50%)	<4 mm
	DBBM alone	6				
Tatullo et al., 2012 [15]	L-PRF + DBBM4	30	NA		12 (20%)	<5 mm
	DBBM alone	30				
Zhang et al., 2012 [14]	L-PRF + DBBM4	5	43.5	NA	8 (80%)	<5 mm
	DBBM alone	5	46.2	NA		

**Table 3:** Summary characteristics of the included studies. L-PRF: leukocyte-platelet-rich fibrin; DBBM: deproteinized bovine bone mineral; ISQ: Implant Stability Quotient

Study	Study design	Sample size	Intervention	Control	Outcomes	L-PRF methodology	Follow-up (month)
Pichotano et al., 2019 [6]	Randomized controlled trial	12	L-PRF + DBBM4	DBBM alone	New bone formation/residual bone/ISQ/reduction in the mean graft volume/amount of fibrous tissue in the maxillary sinus cavity	Dohan technique	8
de Almeida Malzoni et al., 2023 [7]	Randomized controlled trial	36	(1) L-PRF + DBBM4; (2) L-PRF + DBBM8	DBBM alone	New bone formation/residual bone/ISQ/amount of fibrous tissue	Dohan technique	8
Nizam et al., 2018 [8]	Randomized controlled trial	22	L-PRF + DBBM4	DBBM alone	New bone formation/residual bone/contact length bone to bone substitute	Dohan technique	6
Zhang et al., 2012 [14]	Randomized controlled trial	10	L-PRF + DBBM4	DBBM alone	New bone formation/residual bone/contact length bone to bone substitute	Choukroun	6
Tatullo et al., 2012 [15]	Randomized controlled trial	60	L-PRF + DBBM4	DBBM alone	Medullary spaces/osteoid borders/trabecular bone	Choukroun	6

Quality Assessment of the Included RCTs

**Figure 2:**
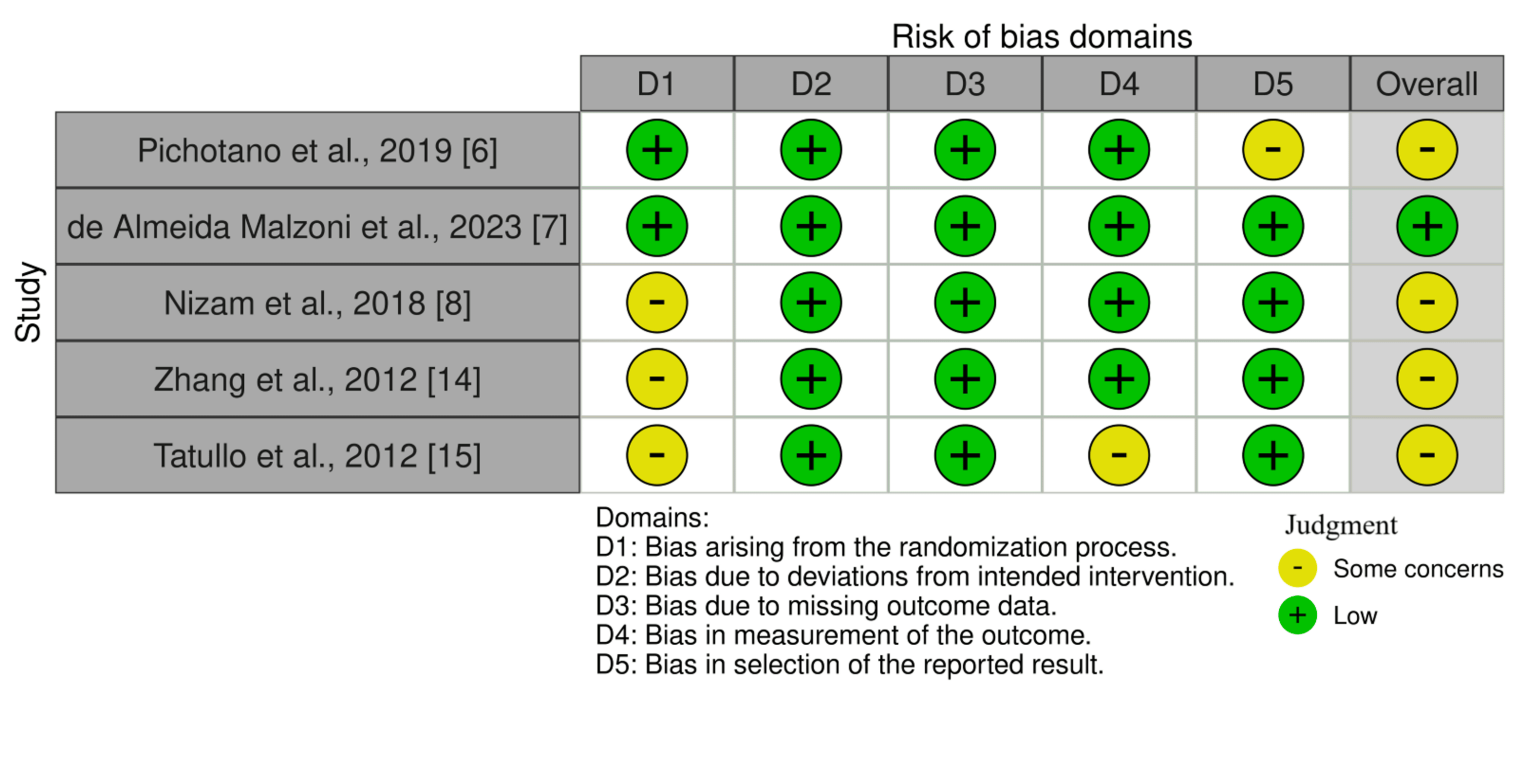
Risk of bias assessment using the ROB-2 tool for RCTs. ROB-2: Risk of Bias 2; RCT: randomized controlled trial

The risk of bias in the included RCTs was assessed using the Cochrane ROB-2 tool. Four studies were rated as having “some concerns,” primarily due to issues related to the randomization process and outcome measurement. Only one study was judged to have an overall low risk of bias. A summary of the risk of bias assessment is presented in Figure 2.

Outcomes

**Figure 3:**
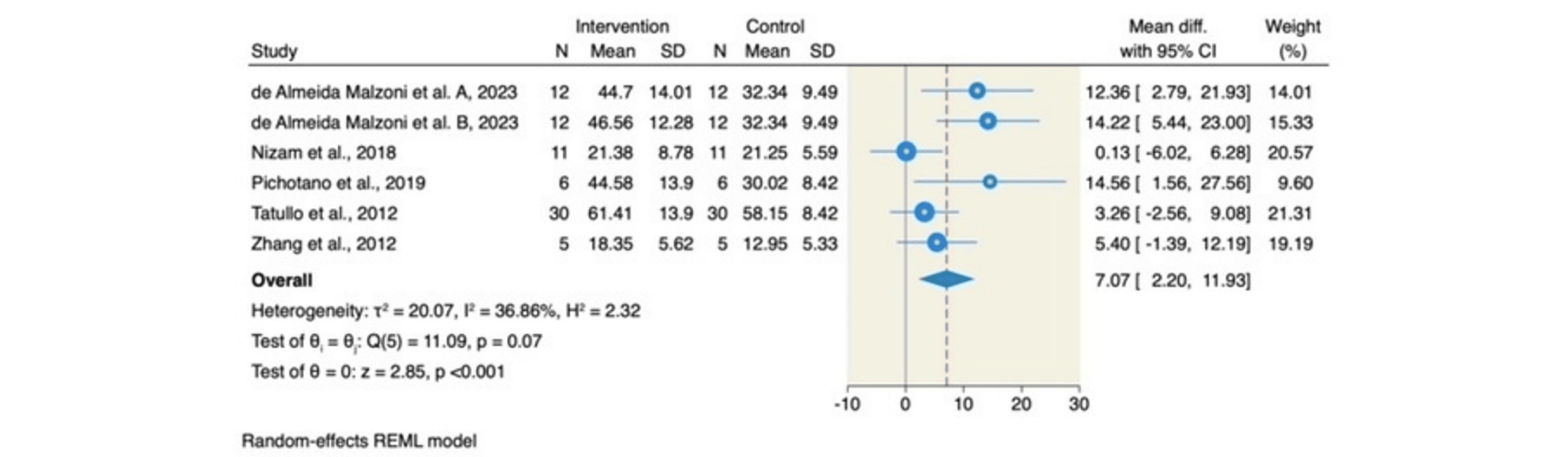
Forest plot of the percentage of new bone formation (%). Source: References [6-8,14,15] REML: restricted maximum likelihood; SD: standard deviation; CI: confidence interval

All included studies assessed the percentage of newly formed bone. The addition of L-PRF to DBBM was associated with a significant increase in new bone formation compared to DBBM alone (MD = 7.07; 95% CI: 2.20-11.93; p < 0.001; I² = 36.86%, p = 0.07), as illustrated in Figure 3.

**Figure 4:**
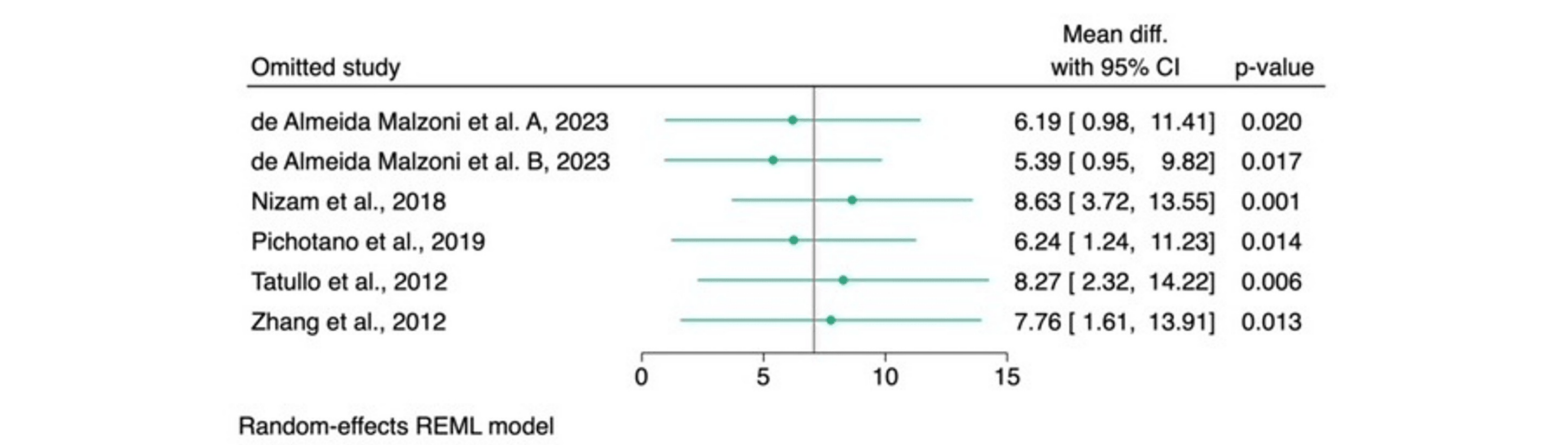
Leave-one-out sensitivity analysis of the percentage of new bone formation (%). Source: References [6-8,14,15] REML: restricted maximum likelihood; CI: confidence interval

Leave-one-out sensitivity analysis revealed that no single study had a disproportionate impact on the overall effect estimate, reinforcing the positive effect of adding L-PRF to DBBM. These results are presented in Figure 4.

**Figure 5:**
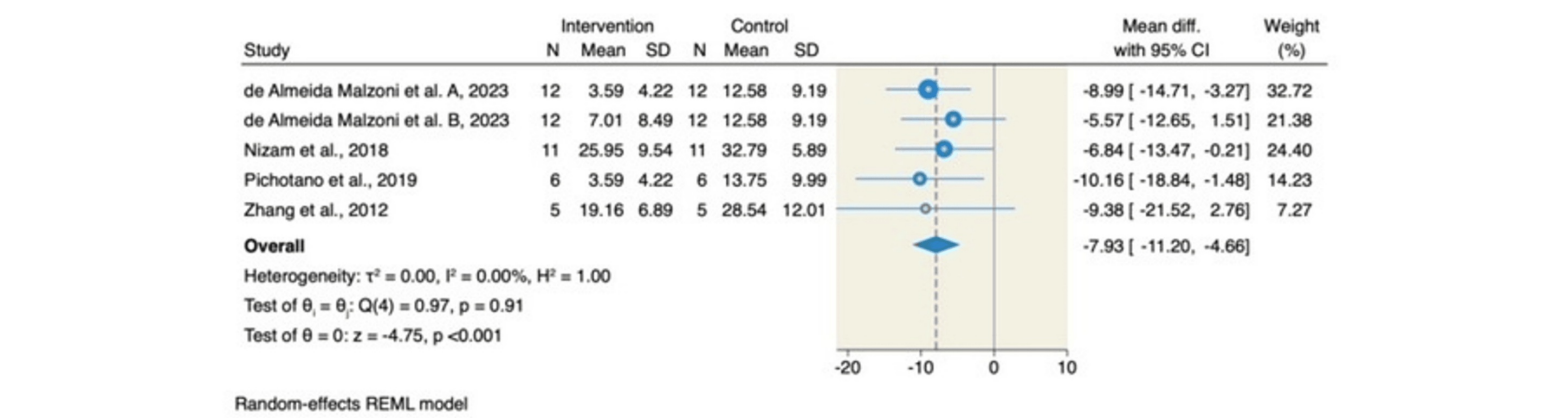
Forest plot of the percentage of residual bone graft (%). Source: References [6-8,14,15] REML: restricted maximum likelihood; SD: standard deviation; CI: confidence interval

Additionally, the combination of L-PRF and DBBM resulted in a significantly lower percentage of residual graft material compared to DBBM alone (MD = -7.93; 95% CI: -11.20 to -4.66; p < 0.001; I² = 0.00%, p = 0.91), as shown in Figure 5.

**Figure 6:**
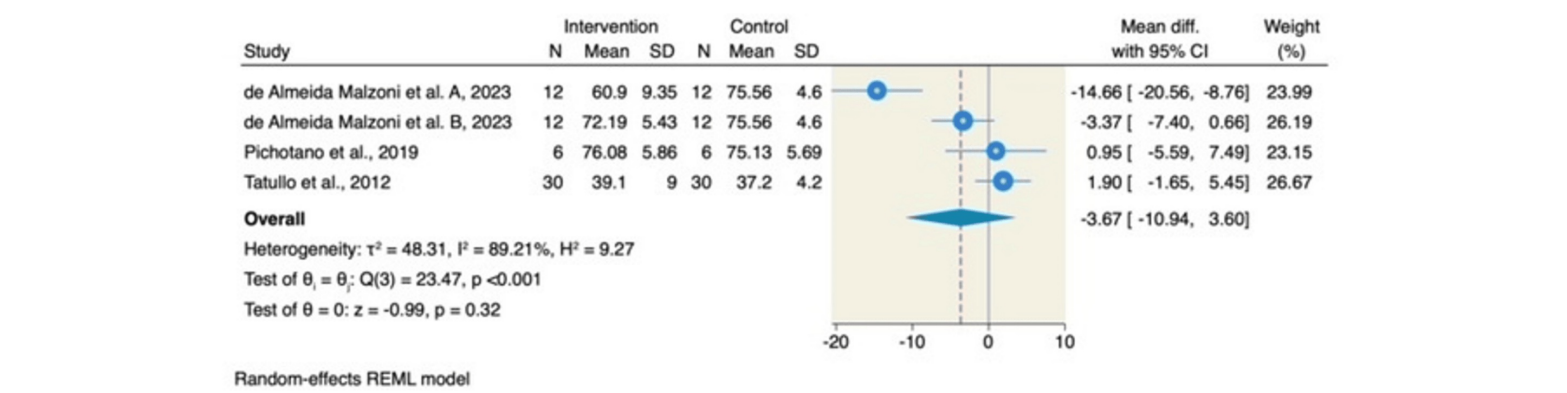
Forest plot of the ISQ at implant loading. Source: References [6-8,14,15] REML: restricted maximum likelihood; SD: standard deviation; CI: confidence interval; ISQ: Implant Stability Quotient

**Figure 7:**
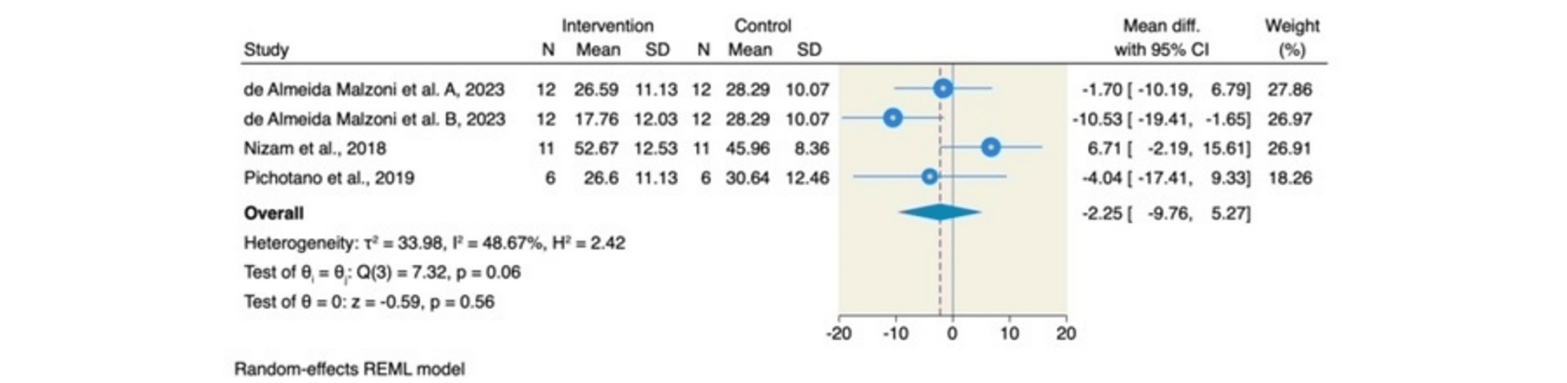
Forest plot of the percentage of soft tissue (%). Source: References [6-8,14,15] REML: restricted maximum likelihood; SD: standard deviation; CI: confidence interval

On the other hand, no significant differences were observed between the L-PRF with DBBM group and the DBBM-only group in terms of ISQ values at the time of implant loading (MD = -2.25; 95% CI: -9.76 to 5.27; p = 0.56; I² = 48.67%, p = 0.06) or the percentage of soft tissue (MD = -3.67; 95% CI: -10.94 to 3.60; p = 0.32; I² = 89.21%, p < 0.001). These findings are presented in Figures 6, 7, respectively.

Discussion

The present systematic review and meta-analysis of five RCTs involving 140 patients represents the most comprehensive effort to date to evaluate the effectiveness of adding L-PRF to DBBM in maxillary sinus augmentation. Our findings demonstrated that the adjunctive use of L-PRF significantly enhanced new bone formation and reduced residual graft material compared to the use of DBBM alone. However, no significant differences were observed in the percentage of soft tissue or the ISQ at the time of implant loading between the two groups.

The potential of L-PRF to accelerate bone regeneration in sinus augmentation has been previously reported in several clinical trials [8,14,16]. For instance, Choukroun et al. [16] evaluated the combination of L-PRF and DBBM and observed a reduction in healing duration without compromising bone neoformation after four months of healing. Similarly, Zhang et al. [14] reported a 1.4-fold increase in new bone formation and a 0.5-fold reduction in residual graft material in the L-PRF group compared to DBBM alone, as confirmed through histomorphometric analysis.

The significant enhancement in bone regeneration observed in our analysis may be attributed to the biological properties of L-PRF. Its dense fibrin matrix serves as a scaffold that promotes cellular migration, while the slow and sustained release of cytokines and growth factors-such as VEGF, PDGF, and TGF-β1-supports angiogenesis, osteoblastic differentiation, and tissue repair [17]. Notably, the unique slow polymerization process during centrifugation results in a flexible, three-dimensional fibrin network, which enhances the entrapment and prolonged release of growth factors [18]. These characteristics distinguish L-PRF from other platelet concentrates and may explain its superior regenerative potential [19].

Nevertheless, some studies have reported contrasting outcomes. Nizam et al. [8], for example, found no significant difference in new bone formation between the L-PRF + DBBM group and the DBBM-alone group after a six-month healing period. Such variability in results may be due to differences in the preparation and application of L-PRF, including variations in centrifugation protocols (e.g., relative centrifugal force and time), the type of centrifuge and collection tubes used, the volume of blood drawn, and the number of L-PRF membranes applied [20]. Additionally, differences in surgical techniques and healing durations across studies may also contribute to the observed heterogeneity [21].

Our analysis has several limitations that should be considered. First, there was variability in the reported healing durations across studies, and not all trials specified the initial sinus volume or the precise amount of grafting material used. These data could provide critical insight into the resorption dynamics and the extent of new bone formation. Furthermore, although the included RCTs were generally of moderate to good quality, four were rated as having “some concerns” in the risk of bias assessment, particularly regarding randomization procedures and outcome assessment.

To strengthen the evidence base, future research should prioritize well-designed, multicenter RCTs with standardized protocols for L-PRF preparation and application. Studies should report key procedural variables such as graft volume, sinus anatomy, and healing duration in detail. Moreover, incorporating long-term follow-up data and clinically relevant endpoints-such as implant survival, patient-reported outcomes, and cost-effectiveness, helps clarify the full scope of benefits associated with L-PRF in sinus augmentation. Harmonization of histomorphometric evaluation methods is also essential to facilitate more reliable comparisons across future studies.

## Conclusions

In conclusion, this systematic review and meta-analysis of five RCTs involving 140 patients demonstrated that the combination of L-PRF with DBBM significantly enhances new bone formation and reduces the residual graft material compared to DBBM alone in maxillary sinus augmentation. These findings support the adjunctive use of L-PRF in sinus lift procedures to potentially enhance outcomes and reduce healing times. However, further high-quality, long-term RCTs with standardized protocols-particularly regarding healing time and the amount of graft material used-are needed to validate and strengthen these findings.
